# Supramolecular catalyst functions in catalytic amount: cucurbit[8]uril accelerates the photodimerization of Brooker’s merocyanine†
†Electronic supplementary information (ESI) available. See DOI: 10.1039/c7sc04125j


**DOI:** 10.1039/c7sc04125j

**Published:** 2017-10-13

**Authors:** Yuetong Kang, Xiaoyan Tang, Hongde Yu, Zhengguo Cai, Zehuan Huang, Dong Wang, Jiang-Fei Xu, Xi Zhang

**Affiliations:** a Key Lab of Optoelectronics and Molecular Engineering , Department of Chemistry , Tsinghua University , Beijing 100084 , China . Email: xujf@mail.tsinghua.edu.cn ; Email: xi@mail.tsinghua.edu.cn

## Abstract

Supramolecular catalysis aims to modulate chemical reactions on both selectivity and rate by taking advantage of supramolecular chemistry. However, due to the effect of product inhibition, supramolecular catalysts are usually added in stoichiometric amounts. Herein, we report a supramolecular catalysis system in which 1% of the supramolecular catalyst, cucurbit[8]uril, is able to significantly accelerate the photodimerization of Brooker’s merocyanine. This catalytic process is realized in a cyclic manner because the photodimerized product can be spontaneously replaced by monomeric reactants *via* competitive host–guest complexation. Thus, a catalytic amount of cucurbit[8]uril is sufficient to accomplish photodimerization within 10 min. This line of research will enrich the field of supramolecular catalysis and allow the development of more efficient catalytic systems.

## Abstract 2

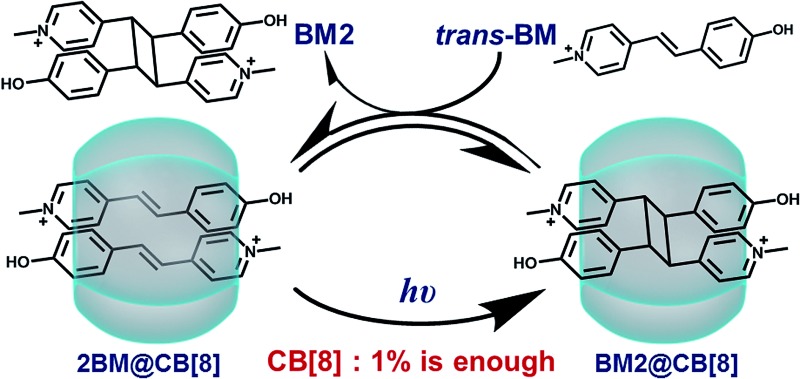
As low as 1% content of a supramolecular catalyst, cucurbit[8]uril, is sufficient to accomplish the photodimerization of Brooker’s merocyanine within 10 minutes.

## Introduction

Supramolecular catalysis aims to modulate chemical reactions on both selectivity and rate by taking advantage of supramolecular chemistry, which is usually towards and even beyond the fine imitation of natural enzymatic catalysis.^1^–^12^ In the past few decades, various host molecules have been developed to become supramolecular catalysts, such as cyclodextrins and supramolecular cages.^13^–^16^ Among them are cucurbit[*n*]urils (CB[*n*]s), a series of macrocycles with varied units of glycouril moieties.^17^,^18^ Various kinds of guest compound can be encapsulated into a CB[*n*] cavity in terms of size fit and complementary interactions^19^–^23^ since CB[*n*]s provide their guest molecules with a specific microenvironment which differs from that of the bulk solution. Basically, CB[*n*]s can influence the reaction activity of the guest molecules in two different ways.^24^–^35^ On the one hand, CB[*n*]s can shield whole guest molecules or their specific moieties from external reagents.^36^,^37^ On the other hand, CB[*n*]s can act as nanoreactors for promoting the guest molecules to undergo certain reactions.^38^–^42^

Generally, product inhibition has always been regarded as a vital drawback in many supramolecular catalysis systems. If the products have a stronger affinity with the supramolecular catalysts than the reactants, the products would firmly occupy the active sites to hinder the reactants from further effective binding, thus deactivating the supramolecular catalysts. Therefore, in order to achieve high conversion of reactants, especially when they are involved in a combination reaction, supramolecular catalysts are usually employed in stoichiometric or even excess amounts.

**Scheme 1:**
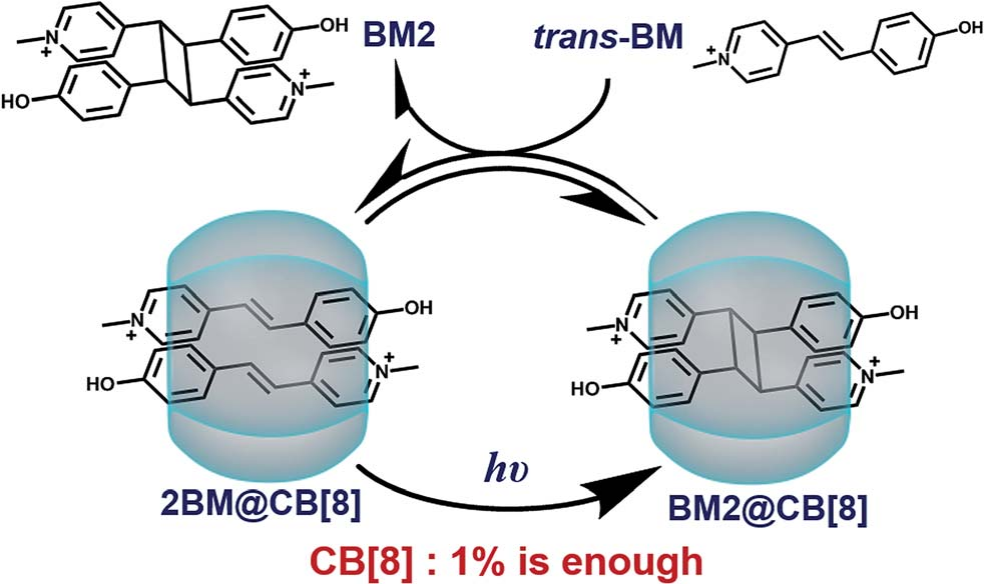
Schematic illustration of the cucurbit[8]uril-based supramolecular catalytic cycle.

In this article, we will report an example in which the addition of a catalytic amount of a supramolecular catalyst is achieved. As shown in Scheme 1, the reactant, Brooker’s merocyanine (BM),^43^–^46^ can be encapsulated into the cavity of CB[8] in a molar ratio of 2 : 1, where photodimerization of BM can be significantly and unidirectionally promoted. Then, the dimerized product can be replaced by another pair of reactants *via* competitive complexation, thus reviving the active site and rendering the catalytic process in a cyclic manner. Therefore, for the first time we have demonstrated that a catalytic amount of CB[8] is sufficient for the effective acceleration of the photodimerization of BM.

## Results and discussions

**Fig. 1:**
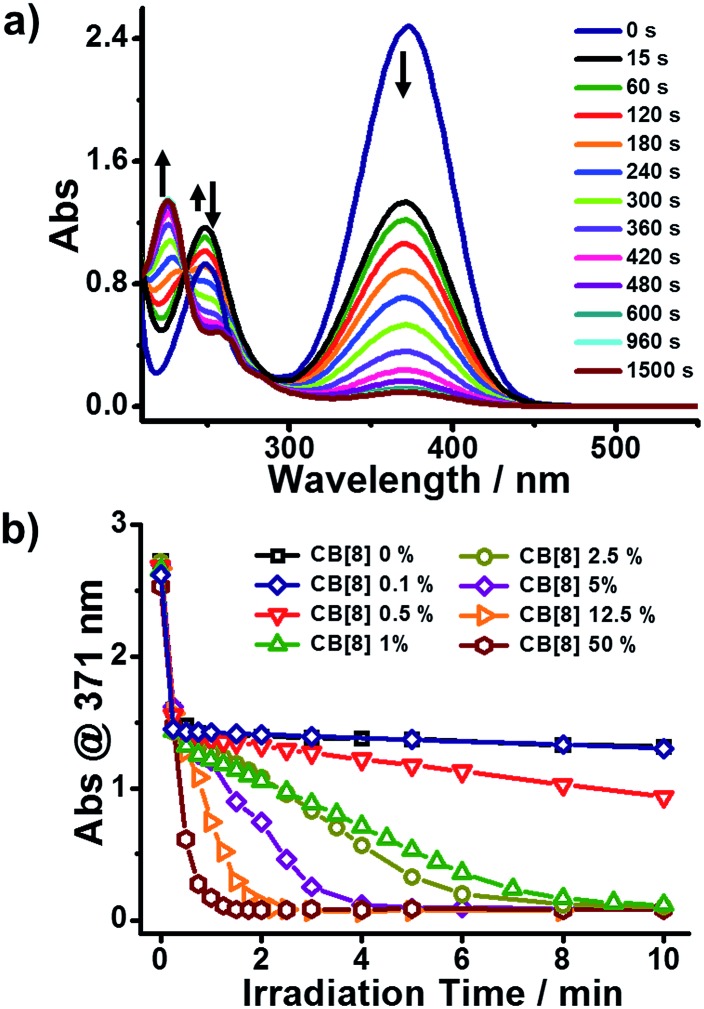
(a) Spectra of the BM : CB[8] = 2 : 0.02 (1% CB[8]) solution under UV irradiation. (b) Diagram of Abs@371 *versus* irradiation time of 0.5 mM BM solutions with varied content of CB[8] under UV irradiation.

The photoreaction of BM in the presence of a varied CB[8] content was monitored by UV-Vis spectroscopy. As shown in Fig. 1a, there was an absorption peak around 371 nm (Abs@371), ascribed to the π–π* transition of *trans*-BM, which was supported by computational data (ESI, Fig. S2†). Abs@371 decreased abruptly during the first 15 s under UV irradiation because of the photoisomerization from *trans*-BM to *cis*-BM. Concomitantly, the absorbance at 250 nm increased during the first 15 s, which was mainly ascribed to the *cis*-BM. With an extension of the UV irradiation, there appeared a new absorption peak around 226 nm, suggesting that the photodimerization occurred and converted BM into a dimer (BM2). Abs@371 was used to monitor the kinetics of the photoreactions of BM with the varied CB[8] content. As shown in Fig. 1b, with the addition of 50% of CB[8] (rendering the molar ratio of BM : CB[8] as 2 : 1), it took 90 s to complete the photodimerization. With the decreasing content of CB[8], it would take more time for the photodimerization. But interestingly, the photoreaction could still be completed within 10 min when the content of CB[8] was as low as 1% (BM : CB[8] is 2 : 0.02), corresponding to a TON of around 50. Therefore, this indicates that a catalytic amount of CB[8] is sufficient to accelerate the photoreaction of Brooker’s merocyanine.

**Fig. 2:**
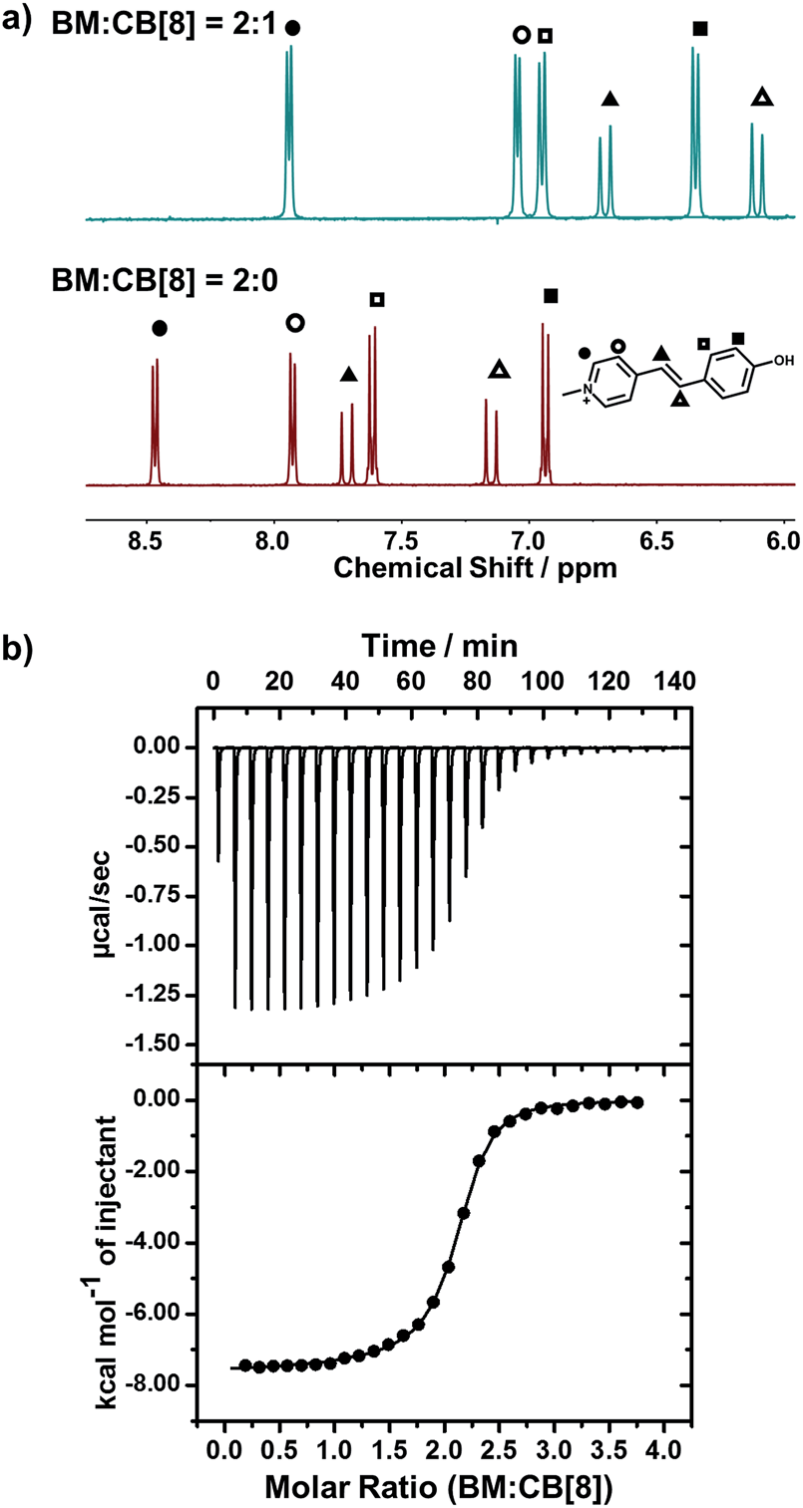
(a) ^1^H-NMR spectra of BM : CB[8] = 2 : 0 and BM : CB[8] = 2 : 1; (b) ITC data for the titration of BM to CB[8].

We employed NMR and isothermal titration calorimetry (ITC) to confirm the complexation between CB[8] and *trans*-BM, which is a prerequisite for efficient catalysis. As shown in the ^1^H NMR spectra (Fig. 2a), the four sets of aromatic protons and the two sets of alkene protons shifted upfield after the introduction of CB[8], indicating that BM was encapsulated by CB[8]. The host–guest complexation of CB[8] and BM was further quantitatively confirmed by ITC. As shown in Fig. 2b, BM and CB[8] formed a ternary complex in a molar ratio of 2 : 1. By fitting the ITC curve using a sequential binding model, the overall binding constant *K*_a_ was estimated to be 8.50 × 10^11^ M^–2^. Using the first binding constant *K*_a1_ of 2.95 × 10^5^ M^–1^ and the second binding constant *K*_a2_ of 2.88 × 10^6^ M^–1^, the cooperativity index was calculated to be 39.0.^47^ This indicates that the homoternary complexation exhibits positive cooperativity, *i.e.* incorporation of the first BM favors the incorporation of the second BM in the cavity of CB[8], which can benefit the photodimerization within the cavity of CB[8].

**Fig. 3:**
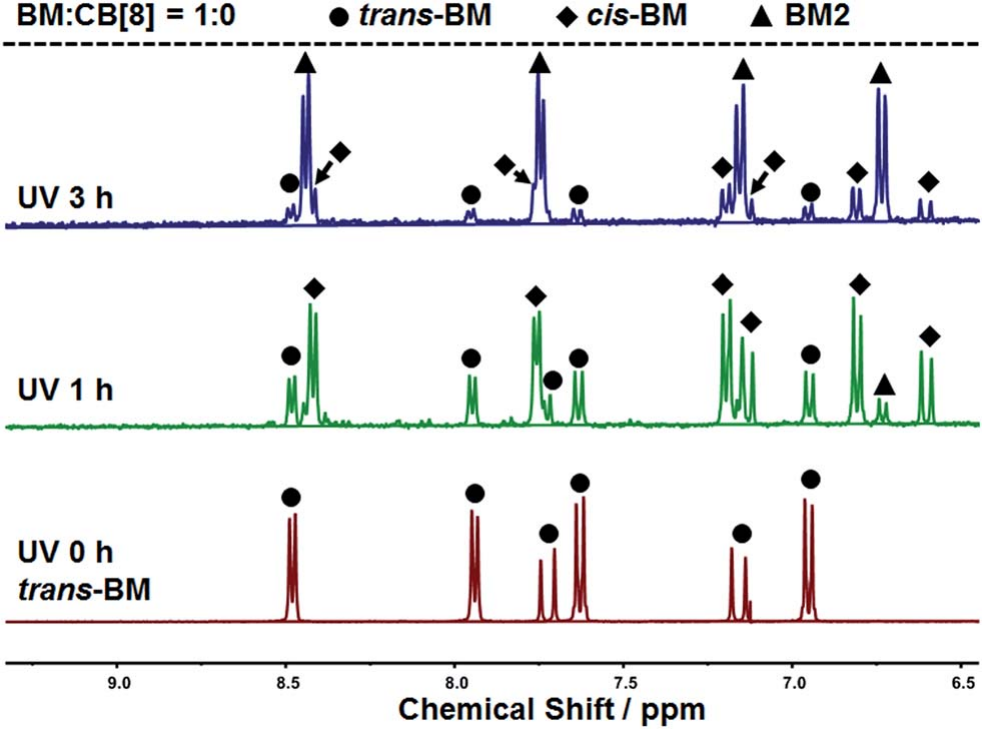
^1^H NMR spectra of BM solutions with varied irradiation time.

**Fig. 4:**
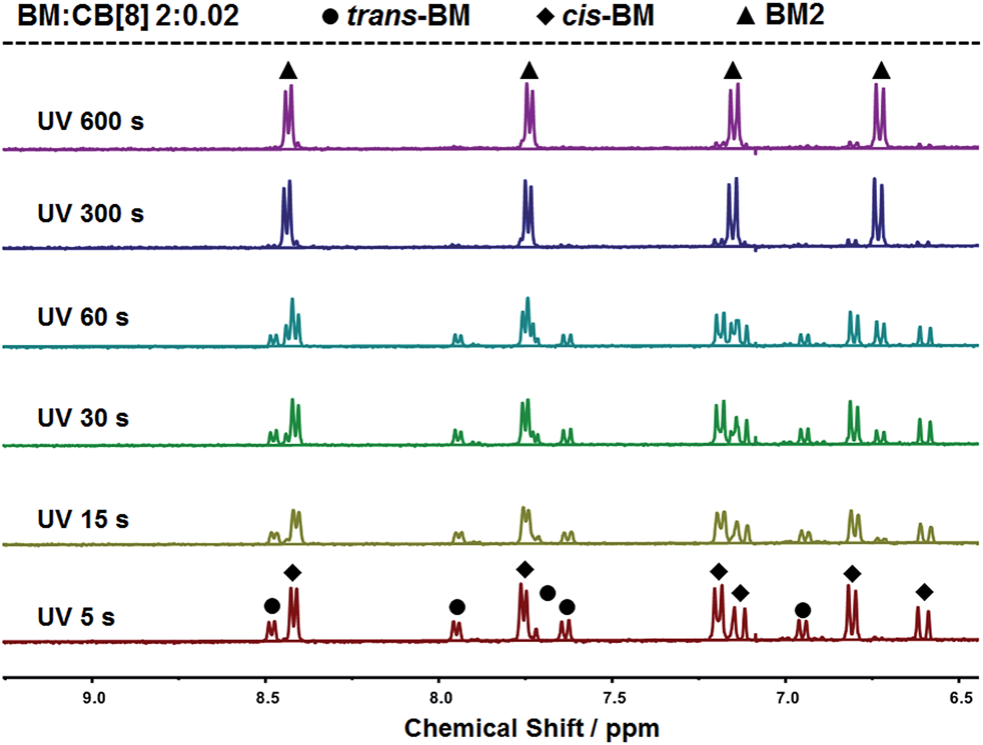
^1^H NMR spectra of BM solutions containing 1% CB[8] with varied irradiation time.

The photoreaction of BM was studied by NMR. BM originally existed in the *trans*-form before UV irradiation, as shown in Fig. 3. BM was photoisomerized from the *trans*-form to the *cis*-form under UV irradiation at the beginning, and there coexisted *trans*-form BM and *cis*-form BM in a molar ratio of about 1 : 2. With further extension of UV irradiation, but without the addition of CB[8], BM could be gradually photodimerized. At 3 hours of UV irradiation, about 62% of BM was converted to the dimer. With the addition of CB[8], the photodimerization was significantly accelerated. As shown in Fig. 4, when the content of CB[8] was 1%, almost 100% of BM was converted into the dimer within 10 min. The non-stoichiometric and fast conversion of BM by CB[8] was also confirmed by ^13^C NMR as shown in Fig. S3.† This may suggest that *trans*-BM was photodimerized in the cavity of CB[8], accompanying the photoisomerization from *cis*-BM to *trans*-BM in the bulk, and then the replacement of the dimer by the new *trans*-BM. Therefore, both *cis*-BM and *trans*-BM were consumed during the UV irradiation and finally converted into the dimer.

**Fig. 5:**
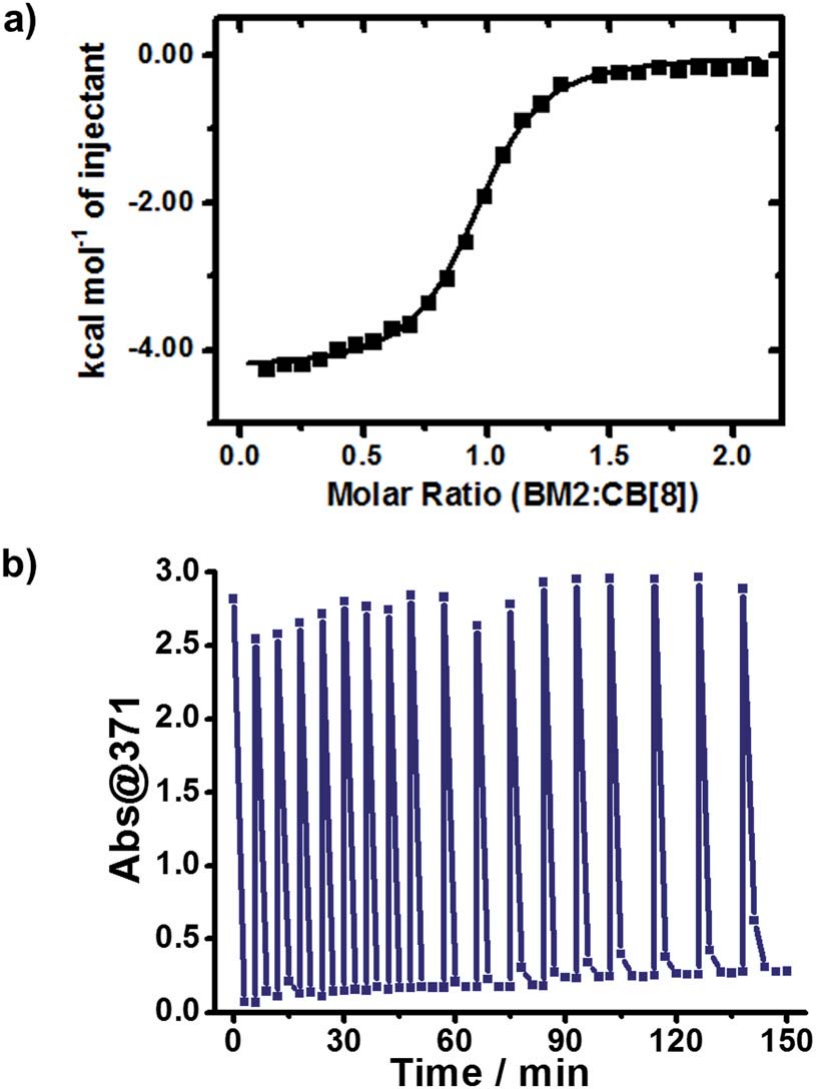
(a) ITC data for the titration of the dimeric product (BM2) to CB[8]. (b) Diagram of Abs@371 *versus* irradiation time with continuous addition of BM for 18 cycles (initial concentration of BM was 0.5 mM).

According to the above assumption, the effective detachment of dimer from CB[8] should be the crucial step in the catalytic cycle. To confirm this assumption, we relied on ITC to characterize the host–guest complexation of the dimer (BM2) and CB[8]. BM2 and CB[8] formed a binary complex, with a binding constant *K*_a_(BM2 : CB[8]) of 2.20 × 10^6^ M^–1^ (Fig. 5a). Considering the competitive complexation between BM and BM2 towards CB[8], the equilibrium constant (*K*_eq_) was calculated to be 3.86 × 10^5^ M^–1^ (Fig. S4†), which meant that the replacement of BM2 by BM was a spontaneous process, and the unreacted BM was more likely to bind with CB[8] than BM2. This result showed that the revival of the CB[8] cavity was not only permitted due to the dynamic nature of the noncovalent host–guest complexation, but was also reinforced by the competitive effect of the unreacted BM against the dimerized product. Therefore, the sustained catalytic activity of CB[8] is achieved by effective detachment of the dimerized product.

We wondered if CB[8] could act as a catalyst to convert the reactant continuously. To answer this question, we refuelled the photoreacted solution with the same amount of BM as its original content, and then irradiated the refuelled sample with UV light. As shown in Fig. 5b, the refuelling-irradiating process could be repeated many times. There was almost no sign of the declination of the catalytic activity of CB[8] even when these refuelling-irradiating experiments were extended to 18 rounds. Therefore, this consolidates that CB[8] is a supramolecular catalyst for this system, and CB[8] preserves its catalytic activity during the photocatalytic processes.

## Conclusion

In summary, we have demonstrated that CB[8] can significantly promote the photodimerization of Brooker’s merocyanine. We consider that the catalytic activity of CB[8] to BM can be ascribed to both the reactivity enhancement of BM within the CB[8] cavity, and the higher binding affinity of reactant than product towards CB[8]. For this system, we anticipate that the distance between the two positive charges of the dicationic product BM2 is larger than the optimistic distance for CB[8] to bind, thus rendering CB[8] more likely to bind two new BM molecules and encycle the catalytic process consequently.

Based on the above anticipation, we are trying to extend this method to other photodimerizable reactants such as monocationic derivatives of stilbene, naphthalene, anthracene and coumarin. Besides, a similar method may be extended to other photochemical reactions as well, and such a field could be further developed by cucurbituril derivatives with modified structures. In addition, CB[8]-promoted photoreactions are likely to be utilized for fabricating covalently-attached polymers or supramolecular polymers with controlled molecular weight or even sequential structures. It is anticipated that this work will enrich the library of supramolecular catalysis systems.

## Conflicts of interest

There are no conflicts to declare.

## Supplementary Material

Supplementary information

## References

[cit1] Lehn J.-M., in Supramolecular Chemistry, Wiley VCH Verlag GmbH & Co. KGaA, 1995, pp. 55–67.

[cit2] Breslow R., Overman L. E. (1970). J. Am. Chem. Soc..

[cit3] Breslow R. (1995). Acc. Chem. Res..

[cit4] Kang J., Rebek J. (1997). Nature.

[cit5] Ramamurthy V. (2015). Acc. Chem. Res..

[cit6] Hastings C. J., Pluth M. D., Bergman R. G., Raymond K. N. (2010). J. Am. Chem. Soc..

[cit7] Vriezema D. M., Aragones M. C., Elemans J. A. A. W., Cornelissen J. J. L. M., Rowan A. E., Nolte R. J. M. (2005). Chem. Rev..

[cit8] Raynal M., Ballester P., Vidal-Ferran A., van Leeuwen P. W. (2014). Chem. Soc. Rev..

[cit9] Sokolov A. N., Bucar D. K., Baltrusaitis J., Gu S. X., MacGillivray L. R. (2010). Angew. Chem., Int. Ed..

[cit10] Tang Y., Zhou L., Li J., Luo Q., Huang X., Wu P., Wang Y., Xu J., Shen J., Liu J. (2010). Angew. Chem., Int. Ed..

[cit11] Dydio P., Reek J. N. H. (2014). Chem. Sci..

[cit12] Cussó O., Giuliano M. W., Ribas X., Miller S. J., Costas M. (2017). Chem. Sci..

[cit13] Zhang D., Jamieson K., Guy L., Gao G., Dutasta J. P., Martinez A. (2017). Chem. Sci..

[cit14] Yoshizawa M., Tamura M., Fujita M. (2006). Science.

[cit15] Pluth M. D., Bergman R. G., Raymond K. N. (2007). Science.

[cit16] Yang C., Ke C., Liang W., Fukuhara G., Mori T., Liu Y., Inoue Y. (2011). J. Am. Chem. Soc..

[cit17] Kim J., Jung I.-S., Kim S.-Y., Lee E., Kang J.-K., Sakamoto S., Yamaguchi K., Kim K. (2000). J. Am. Chem. Soc..

[cit18] Day A. I., Blanch R. J., Arnold A. P., Lorenzo S., Lewis G. R., Dance I. (2002). Angew. Chem., Int. Ed..

[cit19] Barrow S. J., Kasera S., Rowland M. J., del Barrio J., Scherman O. A. (2015). Chem. Rev..

[cit20] Kaifer A. E. (2014). Acc. Chem. Res..

[cit21] Isaacs L. (2014). Acc. Chem. Res..

[cit22] Ni X.-L., Chen S., Yang Y., Tao Z. (2016). J. Am. Chem. Soc..

[cit23] Li J., Zeng Y., Zhang X., Yu T., Chen J., Li Y. (2015). Acta Chim. Sin..

[cit24] Sashuk V., Butkiewicz H., Fialkowski M., Danylyuk O. (2016). Chem. Commun..

[cit25] Wang R., Yuan L., Macartney D. H. (2006). Chem. Commun..

[cit26] Yang H., Ma Z., Wang Z., Zhang X. (2014). Polym. Chem..

[cit27] Koner A. L., Nau W. M. (2007). Supramol. Chem..

[cit28] Li S., Wyman I. W., Wang C., Wang Y., Macartney D. H., Wang R. (2016). J. Org. Chem..

[cit29] Assaf K. I., Nau W. M. (2015). Chem. Soc. Rev..

[cit30] Tian J., Zhou T. Y., Zhang S. C., Aloni S., Altoe M. V., Xie S. H., Wang H., Zhang D. W., Zhao X., Liu Y., Li Z. T. (2014). Nat. Commun..

[cit31] Wang X. Q., Lei Q., Zhu J. Y., Wang W. J., Cheng Q., Gao F., Sun Y. X., Zhang X. Z. (2016). ACS Appl. Mater. Interfaces.

[cit32] Masson E., Shaker Y. M., Masson J.-P., Kordesch M. E., Yuwono C. (2011). Org. Lett..

[cit33] Lu X., Masson E. (2010). Org. Lett..

[cit34] Mock W. L., Irra T. A., Wepsiec J. P., Manimaran T. L. (1983). J. Org. Chem..

[cit35] Ke C., Strutt N. L., Li H., Hou X., Hartlieb K. J., McGonigal P. R., Ma Z., Iehl J., Stern C. L., Cheng C., Zhu Z., Vermeulen N. A., Meade T. J., Botros Y. Y., Stoddart J. F. (2013). J. Am. Chem. Soc..

[cit36] Yang H., Liu Y., Yang L., Liu K., Wang Z., Zhang X. (2013). Chem. Commun..

[cit37] Ren H., Huang Z., Yang H., Xu H., Zhang X. (2015). ChemPhysChem.

[cit38] Wang R., Yuan L., Macartney D. H. (2006). J. Org. Chem..

[cit39] Pattabiraman M., Natarajan A., Kaanumalle L. S., Ramamurthy V. (2005). Org. Lett..

[cit40] Pemberton B. C., Raghunathan R., Volla S., Sivaguru J. (2012). Chemistry.

[cit41] Gromov S. P., Vedernikov A. I., Kuz’mina L. G., Kondratuk D. V., Sazonov S. K., Strelenko Y. A., Alfimov M. V., Howard J. A. K. (2010). Eur. J. Org. Chem..

[cit42] Zheng L., Sonzini S., Ambarwati M., Rosta E., Scherman O. A., Herrmann A. (2015). Angew. Chem., Int. Ed..

[cit43] Abdel-Kader M. H., Steiner U. (1983). J. Chem. Educ..

[cit44] Brooker L. G. S., Keyes G. H., Heseltine D. W. (1951). J. Am. Chem. Soc..

[cit45] Brooker L. G. S., Keyes G. H., Sprague K. H., Van Dyke R. H., Van Lare E., Van Zandt G., White F. L. (1953). J. Am. Chem. Soc..

[cit46] Nandi L. G., Nicoleti C. R., Bellettini I. C., Machado V. G. (2014). Anal. Chem..

[cit47] Huang Z., Qin K., Deng G., Wu G., Bai Y., Xu J.-F., Wang Z., Yu Z., Scherman O. A., Zhang X. (2016). Langmuir.

