# Supplementary material for: Supramolecular catalyst functions in catalytic amount: cucurbit[8]uril accelerates the photodimerization of Brooker’s merocyanine
Source: Chem Sci. 2017 Oct 13;8(12):8357–61. doi: 10.1039/c7sc04125j (PMC5858745; doi:10.1039/c7sc04125j)
Supplement: Supplementary file 1 [file SC-008-C7SC04125J-s001.pdf]

Electronic Supplementary Material (ESI) for Chemical Science.
This journal is © The Royal Society of Chemistry 2017

Supporting Information

Supramolecular Catalyst Functions in Catalytic Amount: Cucurbit[8]uril Accelerates the Photodimerization of Brooker's Merocyanine

Yuetong Kang, Xiaoyan Tang, Hongde Yu, Zhengguo Cai, Zehuan Huang, Dong Wang, Jiang-Fei Xu and Xi Zhang**

Key Lab of Organic Optoelectronics and Molecular Engineering, Department of Chemistry, Tsinghua University, Beijing 100084, China

E-mail: xujf@mail.tsinghua.edu.cn

xi@mail.tsinghua.edu.cn

SI-1 Experimental Section

Materials:

4-picoline, 4-hydroxybenzyl aldehyde, 4-methoxybenzyl aldehyde and deuterium oxide (D, 99.8%) were purchased from J&K Chemicals. Piperidine was purchased from Beijing Chemical Factory. Isopropanol and other solvents were purchased from Sinopharm Chemical Reagent. Cucurbit[8]uril (CB[8]) was prepared by Dr. Hao Chen from our group. Water was purified by a Milli-Q Integral Water Purification System.

Characterization Methods:

UV-Vis spectra were monitored by a HITACHI U-3010 spectrometer, path length: 2.00 mm. ^{1}H-NMR spectra were obtained by a JEOL JNM ECA400 NMR Spectrometer. The UV irradiation experiments were conducted via irradiating the deoxygenated samples by CEAULIGHT CEL-M500 UV irradiator consisting of a high-pressure mercury lamp integrated with a 365 nm narrow bandpass filter. Isothermal titration calorimetry experiments were conducted on a Microcal VP-ITC apparatus at 298.15 K. The titration schedule was 1 injection of 5 μL and 28 consecutive injections of 10 μL with a 300-second interval between injections. All the samples were prepared with acetate buffer (50 mM, pH 5.0)

Synthesis of Brooker's Merocyanine (BM):

The synthesis route of BM is shown in Figure S1.

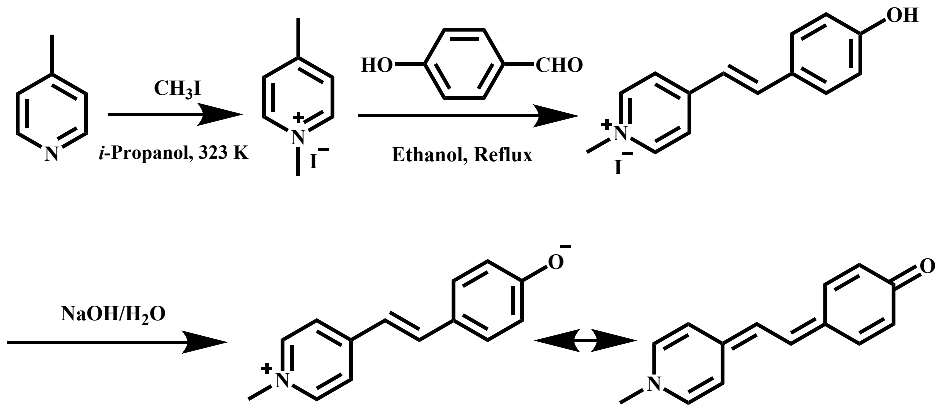

Figure S1. The synthesis route of BM.

1,4-dimethylpyridinium iodide: 0.94 ml methyl iodide was added to the solution of 1.5 ml 4-picoline and 10 ml isopropyl alcohol. The mixture was heated to about 50℃

and the exothemic reaction occurred simultaneously, resulting in a white precipitate. The solid was filtered and washed with isopropyl alcohol.

1-methyl-4-[2-(4-hydroxyphenyl)ethenyl]pyridinium iodide: 2.1 g 4-hydroxybenzyl aldehyde and 1.8 g 1,4-dimethylpyridinium iodide were added to the solution of 15 ml ethanol and 0.2 ml piperidine, and heated to reflux overnight. The resultant solution was concentrated to approximately 5 ml by vacuum rotary evaporation, and was added dropwise to 40 ml diethyl ether. After being filtered off, the red precipitate (compound 2) was washed several times by diethyl ether.

BM: 1-methyl-4-[2-(4-hydroxyphenyl)ethenyl]pyridinium iodide was recrystallized in 62.5 mM NaOH aqueous solution. The resultant solid (BM), appearing as dark purple crystals, was filtered off and dried at 318 K *in vacuo*. ^{1}H NMR (400 MHz, D_2O, pH 5.0): δ 8.48 (d, *J* = 6.8 Hz, 2H), δ 7.94 (d, *J* = 6.8 Hz, 2H), δ 7.72 (d, *J* = 16.3 Hz, 1H), δ 7.63 (d, *J* = 8.7 Hz, 2H), δ 7.16 (d, *J* = 16.3 Hz, 1H), δ 4.23 (s, 3H). ^{13}C NMR (150 MHz, D_2O, pH 5.0): δ 158.10, 153.89, 144.11, 140.75, 130.28, 127.60, 123.42, 120.40, 116.10, 49.56.

SI-2 Supplementary Figures

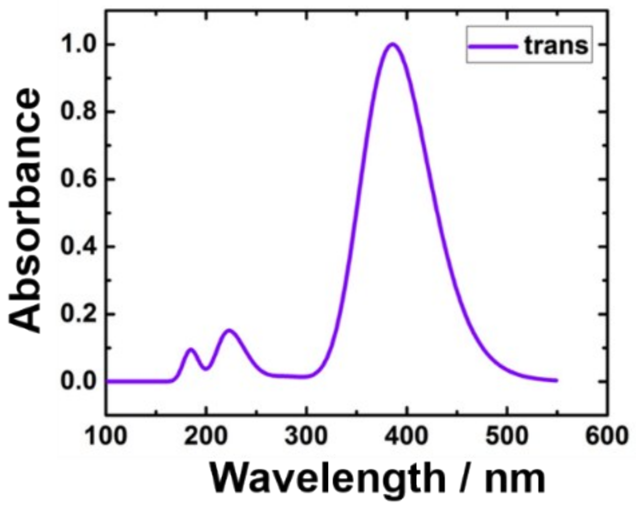

Figure S2. Theoretical UV-Vis spectrum of trans-BM (TDDFT, CAM-B3LYP).

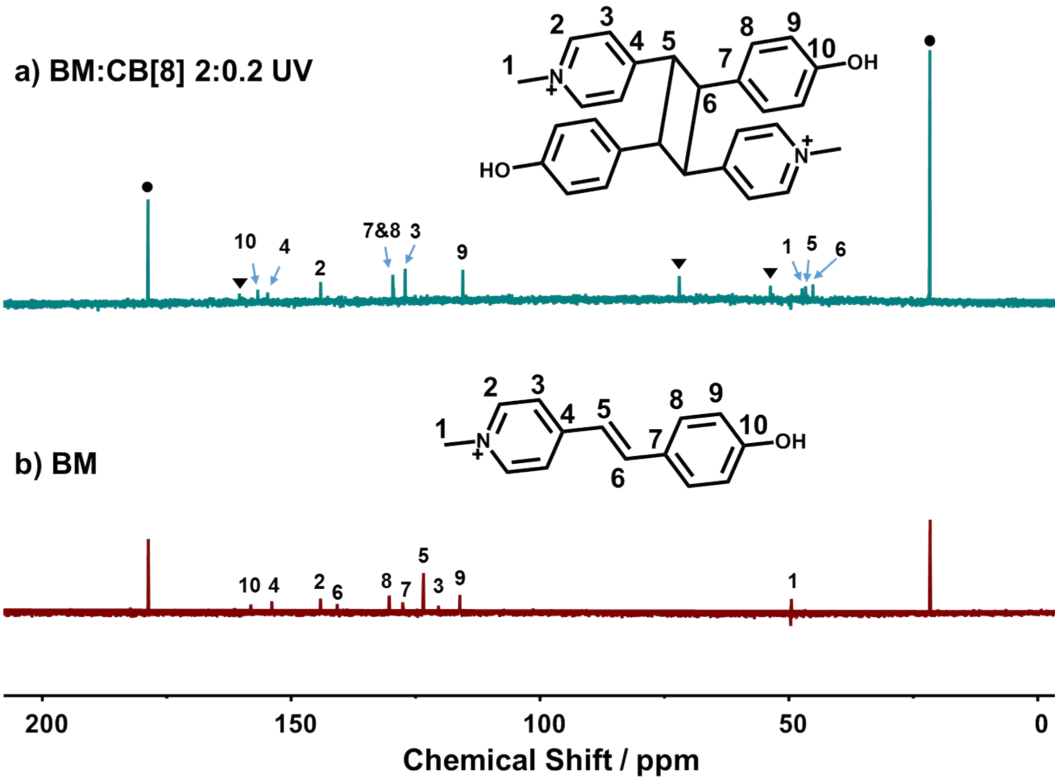

Figure S3. ^{13}C NMR spectra of a) 4 mM BM solution and b) 4 mM BM solution with 10% CB[8] after UV irradiation. Round dots refer to the 2 signals of the acetate anion, and the reverse triangular dots refer to the 3 signals of CB[8].

$$2\text{BM} + (\text{BM2@CB[8]}) \rightleftharpoons (2\text{BM@CB[8]}) + \text{BM2}$$

$$K_a = \frac{[(2\text{BM@CB[8]})] \times [\text{BM2}]}{[(\text{BM2@CB[8]})] \times [\text{BM}]^2} = \frac{K_a\,(\text{BM:CB[8]})}{K_a\,(\text{BM2:CB[8]})} = 3.86 \times 10^5\ \text{M}^{-1}$$

Figure S4. The calculation on the equilibrium constant (K_{eq}) of the competitive host-guest complexation process.
